# Health-Related Behaviors by Urban-Rural County Classification — United States, 2013

**DOI:** 10.15585/mmwr.ss6605a1

**Published:** 2017-02-03

**Authors:** Kevin A. Matthews, Janet B. Croft, Yong Liu, Hua Lu, Dafna Kanny, Anne G. Wheaton, Timothy J. Cunningham, Laura Kettel Khan, Ralph S. Caraballo, James B. Holt, Paul I. Eke, Wayne H. Giles

**Affiliations:** 1 Division of Population Health, National Center for Chronic Disease Prevention and Health Promotion, CDC; 2 Division of Nutrition, Physical Activity, and Obesity, National Center for Chronic Disease Prevention and Health Promotion, CDC; 3 Office on Smoking and Health, National Center for Chronic Disease Prevention and Health Promotion, CDC

## Abstract

**Problem/Condition:**

Persons living in rural areas are recognized as a health disparity population because the prevalence of disease and rate of premature death are higher than for the overall population of the United States. Surveillance data about health-related behaviors are rarely reported by urban-rural status, which makes comparisons difficult among persons living in metropolitan and nonmetropolitan counties.

**Reporting Period:**

2013.

**Description of System:**

The Behavioral Risk Factor Surveillance System (BRFSS) is an ongoing, state-based, random-digit-dialed landline- and cellular-telephone survey of noninstitutionalized adults aged ≥18 years residing in the United States. BRFSS collects data on health-risk behaviors, chronic diseases and conditions, access to health care, and use of preventive health services related to the leading causes of death and disability. BRFSS data were analyzed for 398,208 adults aged ≥18 years to estimate the prevalence of five self-reported health-related behaviors (sufficient sleep, current nonsmoking, nondrinking or moderate drinking, maintaining normal body weight, and meeting aerobic leisure time physical activity recommendations) by urban-rural status. For this report, rural is defined as the noncore counties described in the 2013 National Center for Health Statistics Urban-Rural Classification Scheme for Counties.

**Results:**

Approximately one third of U.S. adults practice at least four of these five behaviors. Compared with adults living in the four types of metropolitan counties (large central metropolitan, large fringe metropolitan, medium metropolitan, and small metropolitan), adults living in the two types of nonmetropolitan counties (micropolitan and noncore) did not differ in the prevalence of sufficient sleep; had higher prevalence of nondrinking or moderate drinking; and had lower prevalence of current nonsmoking, maintaining normal body weight, and meeting aerobic leisure time physical activity recommendations. The overall age-adjusted prevalence of reporting at least four of the five health-related behaviors was 30.4%. The prevalence among the estimated 13.3 million adults living in noncore counties was lower (27.0%) than among those in micropolitan counties (28.8%), small metropolitan counties (29.5%), medium metropolitan counties (30.5%), large fringe metropolitan counties (30.2%), and large metropolitan centers (31.7%).

**Interpretation:**

This is the first report of the prevalence of these five health-related behaviors for the six urban-rural categories. Nonmetropolitan counties have lower prevalence of three and clustering of at least four health-related behaviors that are associated with the leading chronic disease causes of death. Prevalence of sufficient sleep was consistently low and did not differ by urban-rural status.

**Public Health Action:**

Chronic disease prevention efforts focus on improving the communities, schools, worksites, and health systems in which persons live, learn, work, and play. Evidence-based strategies to improve health-related behaviors in the population of the United States can be used to reach the *Healthy People 2020* objectives for these five self-reported health-related behaviors (sufficient sleep, current nonsmoking, nondrinking or moderate drinking, maintaining normal body weight, and meeting aerobic leisure time physical activity recommendations). These findings suggest an ongoing need to increase public awareness and public education, particularly in rural counties where prevalence of these health-related behaviors is lowest.

## Introduction

Health-related behaviors can extend longevity by preventing chronic disease and injury (*1*). Rural populations face many challenges in adopting these behaviors. The low population density in rural areas makes it difficult to deliver services and provide needed health communications about the benefits of adopting these behaviors. Less than 10% of the health care workforce practice in rural settings. Accessing the health care that is available can be difficult because rural residents have lower incomes and lower rates of health insurance compared with their urban counterparts, and they live farther away from health care resources (*2*).

In a recent study of U.S. adults aged ≥21 years who responded to the 2013 Behavioral Risk Surveillance System (BRFSS) questionnaire, 64% reported sleeping ≥7 hours per day, 82% were current nonsmokers, 63% reported moderate or no alcohol consumption, 32% had normal body weight, and 50% met aerobic leisure time physical activity recommendations; 24% reported four and 6% reported all five health-related behaviors (*3*). The highest estimates of engaging in four or five health-related behaviors clustered in the Pacific and Rocky Mountain states and the lowest estimates clustered in the southern states and along the Ohio River (*3*).

Despite improvements in the overall health of the American people, racial/ethnic minorities and other populations experience disproportionately the prevalence of illness and rates of premature death. These populations are referred to as health disparity populations. The National Institutes of Health also recognizes persons living in rural areas as a health disparity population because their prevalence of disease and rate of premature death are higher than for the overall population of the United States (https://www.nimhd.nih.gov/docs/2009-2013nih_health_disparities_strategic_plan_and_budget.pdf). The Minority Health and Health Disparities Research and Education Act of 2000 (Pub. L. No. 106-525, 114 Stat. 2495) defines a population as a health disparity population if the director of the National Center on Minority Health and Health Disparities, after consultation with the director of the Agency for Healthcare Research and Quality, determines there is a significant disparity in the overall rate of disease incidence, prevalence, morbidity, mortality, or survival in the population as compared with the health status of the general population. As such, racial and ethnic minorities, persons with low socioeconomic status, and persons living in rural areas are designated as health disparity populations.

Rural and urban areas can be defined in many ways (*4*), but few surveillance systems have provided adequate sample sizes to allow comparisons of health-related behaviors in the U.S. population beyond urban versus rural or metropolitan versus nonmetropolitan status (*5*–*9*). The 2013 National Center for Health Statistics (NCHS) Urban-Rural Classification Scheme for Counties uses 2010 census data to assign each U.S. county to one of six (four metropolitan, one micropolitan, and one noncore) urban-rural classes (*10*). NCHS uses noncore and rural interchangeably; noncore counties are defined as the nonmetropolitan counties that are not micropolitan. On the basis of that classification, the majority (85.2%) of the total U.S. population live in metropolitan counties, followed by 8.7% in micropolitan and 6.1% in noncore counties (*10*). Findings in a 2014 report on rural health, which categorized U.S. counties into five urban-rural levels according to a 2006 NCHS classification scheme (*2*) and 2000 U.S. census data, indicated that during 2010–2011, estimated prevalence for cigarette smoking, obesity, and physical inactivity was higher in nonmetropolitan than in metropolitan counties (*2*). In contrast, the prevalence of consuming ≥5 drinks in 1 day did not vary by rural-urban level nationwide but was lower in the nonmetropolitan counties in the South and was higher in nonmetropolitan than in metropolitan counties in the West (*2*).

This report assesses the total number and prevalence of five health-related behaviors by NCHS urban-rural county classification using 2013 BRFSS data. The findings can be used by local health care providers and public health professionals to develop effective strategies for communicating to their patients and constituencies the importance of adopting these healthy behaviors.

## Methods

Information about five selected self-reported health-related behaviors was analyzed from the 2013 BRFSS, which is an annual state-based, random-digit-dialed landline- and cellular-telephone survey of the noninstitutionalized U.S. population aged ≥18 years (https://www.cdc.gov/brfss/annual_data/annual_2013.html). BRFSS data are weighted to represent state populations. The 2013 BRFSS is the most recent year for which the survey questionnaire had questions for both sleep and aerobic physical activity in addition to cigarette smoking, alcohol consumption, and height and weight. Response rates for BRFSS are calculated using standards set by the American Association for Public Opinion Research response rate formula 4 (www.aapor.org/AAPOR_Main/media/publications/Standard-Definitions20169theditionfinal.pdf). The response rate is the number of respondents who completed the survey as a proportion of all eligible and likely eligible persons. In 2013, the median survey response rate for all states and the District of Columbia was 46.4 and ranged from 29.0 to 60.3 (https://www.cdc.gov/brfss/annual_data/2013/pdf/2013_DQR.pdf).

The five selected self-reported health-related behaviors (sufficient sleep, current nonsmoking, nondrinking or moderate drinking, maintaining normal body weight, and meeting aerobic leisure time physical activity recommendations) are included as objectives in *Healthy People 2020* (https://www.healthypeople.gov/2020/topics-objectives). The total number and prevalence of these behaviors were analyzed using the NCHS 2013 Urban-Rural Classification Scheme for Counties, which uses 2010 census population data and the February 2013 Office of Management and Budget designations of metropolitan statistical area (MSA), micropolitan statistical area, or noncore area (*9*). This classification system assigns each county in the United States to one of the six following categories: 1) large central metropolitan (county within an MSA with ≥1 million population that also contains the entire population of the largest principal city in the MSA, has the entire county population contained in the largest principal city, or contains at least 250,000 population of any principal city in the MSA); 2) large fringe metropolitan (county in MSA of ≥1 million population that did not qualify as a large central metropolitan county); 3) medium metropolitan (county in MSA with 250,000–999,999 population); small metropolitan (county in MSA with <250,000 population), micropolitan (county in an area that comprises non-MSA counties but has an urban cluster of 10,000–49,999 population, contains outlying counties that meet specified requirements for commuting to or from central counties, or both); and noncore (county in a nonmetropolitan area that did not qualify as micropolitan and might be thought of as rural) (*9*). Of the 3,143 U.S. counties identified in 2013, 2.1% were large central metropolitan, 11.7% were large fringe metropolitan, 11.9% were medium metropolitan, 11.4% were small metropolitan, 20.4% were micropolitan, and 42.5% were noncore counties (*10*).

One of six levels of urban-rural classification was identified for the county of residence for each respondent. County-level identifiers for BRFSS respondents are not publically available. To match a respondent to their urban-rural class, a special data set with the county identified for each BRFSS respondent was obtained through a data use agreement. Comparisons by urban-rural classification of the prevalence of having four or five health-related behaviors were also assessed by age, sex, race/ethnicity, and self-reported education.

Sufficient sleep was defined for an average sleep duration during a 24-hour period as ≥8 hours for adults aged 18–21 years and ≥7 hours for those aged ≥22 years. Current nonsmokers included adults who never smoked, as well as adults who had smoked at least 100 cigarettes in their lifetime but did not report smoking at the time of the survey; persons who had stopped smoking for health reasons were included. Nondrinking or moderate drinking was defined for adults who drank no alcohol or drank alcohol in moderation during the preceding 30 days. Moderate drinking was defined as drinking up to two alcoholic drinks per day for men and up to one drink per day for women among respondents who also did not report binge drinking (≥5 drinks on one occasion for men or ≥4 drinks for women) or heavy drinking (≥15 drinks per week for men or ≥8 drinks per week for women) during the preceding 30 days (https://health.gov/dietaryguidelines/2015/guidelines/appendix-9). Normal body weight was defined as body mass index of 18.5–24.9 kg/m^2^ that was calculated from self-reported height and weight (https://www.healthypeople.gov/2020/topics-objectives/). Meeting the aerobic leisure time physical activity recommendations was defined as at least 150 minutes per week of moderate leisure time physical activity, 75 minutes per week of vigorous-intensity physical activity, or an equivalent combination of moderate and vigorous physical activity in the preceding 30 days (https://health.gov/paguidelines/guidelines/). Unlike an earlier CDC report on the prevalence of the five health-related behaviors (*1*), analyses were not restricted to respondents aged ≥21 years; therefore, for respondents aged 18–20 years, the nondrinking or moderate drinking measure only included those who reported nondrinking because any alcohol consumption would be illegal. The prevalence of the five health-related behaviors was stratified by sex, age group (18–24, 25–34, 35–44, 45–54, 55–64, 65–74, or ≥75 years), race/ethnicity (non-Hispanic white, non-Hispanic black, Hispanic, American Indian/Alaska Native, Asian, Native Hawaiian/Pacific Islander, multiracial non-Hispanic, or other unspecified race), and educational attainment (less than high school graduate, high school graduate, or some college).

In 2013, BRFSS had 483,865 adult respondents in the 50 states and the District of Columbia. The final sample size was 398,208 (82.4%) adults aged ≥18 years who lived in 3,131 (99.6%) of the 3,143 U.S. counties; the sample size was determined after 2,971 pregnant women, who would be unable to maintain normal body weight, and 85,657 respondents, who had incomplete information for any of the five behaviors or age, were excluded. After assignment of each respondent to an urban-rural classification, sample sizes ranged from 53,000 to 87,000 in each of the six urban-rural categories; approximately 63,000 lived in the micropolitan and noncore counties.

The total number (0−5) of health-related behaviors was calculated for each respondent and unadjusted, weighted distributions were obtained for each urban-rural classification category. The age-adjusted prevalence and 95% confidence interval for each health-related behavior and for having four or five of the behaviors was calculated by urban-rural classification levels and selected characteristics and were adjusted to the 2000 standard population (*11*). For comparisons of prevalence among subgroups, statistical significance (p<0.05) was determined by t-tests. Estimates with relative standard error ≥ 30% or n < 50 were deemed unreliable and are not included. The statistical software program SAS-callable SUDAAN version 11.0.1 (RTI International, Research Triangle Park, North Carolina, USA), which accounts for the complex sampling design of BRFSS, was used for the analyses.

## Results

In 2013, adults aged ≥18 years who were living in noncore counties had the lowest age-adjusted prevalence of current nonsmoking, maintaining normal body weight, and meeting aerobic leisure time physical activity recommendations and the highest prevalence of nondrinking or moderate drinking compared with adults living in metropolitan and micropolitan counties (Table 1). Adults living in micropolitan counties also had lower prevalence of current nonsmoking, maintaining normal body weight, and meeting aerobic leisure time physical activity recommendations and higher prevalence of nondrinking or moderate drinking than those living in metropolitan counties. The lowest prevalence of nondrinking or moderate drinking was reported among adults living in large fringe metropolitan counties and large metropolitan centers. Sufficient sleep did not differ by urban-rural classification. Among adults living in noncore counties, about one third of respondents (35.0%) reported having three of the five health-related behaviors.

**TABLE 1:** Prevalence of five health-related behaviors* among adults aged ≥18 years, by urban-rural status^†^ — Behavioral Risk Factor Surveillance System, United States, 2013

Behaviors	Overall	Large metropolitan center	Large fringe metropolitan	Medium metropolitan	Small metropolitan	Micropolitan	Noncore
	% (95% CI)	% (95% CI)	% (95% CI)	% (95% CI)	% (95% CI)	% (95% CI)	% (95% CI)
**Survey respondents (no.)**	**398,208**	**58,375**	**73,622**	**87,083**	**53,069**	**63,226**	**62,833**
**Estimated adult population (no.)**	**189,995,000**	**55,949,000**	**45,198,000**	**40,091,000**	**17,871,000**	**17,604,000**	**13,282,000**
Current nonsmoking	81.0 (80.7–81.3)	83.9 (83.4–84.5)^¶,^**	82.3 (81.7–82.8)^¶,^**	80.5 (79.9–81.0)^¶,^**	77.5 (76.7–78.2)^¶^	76.5 (75.7–7.3)^¶^	74.9 (74.0–5.9)**
Nondrinking or moderate drinking^§^	62.7 (62.3–63.0)	61.1 (60.4–61.9)^¶,^**	59.9 (59.3–60.6)^¶,^**	63.3 (62.6–63.9)^¶,^**	64.3 (63.5–65.1)^¶,^**	67.3 (66.5–68.1)	68.6 (67.7–69.6)
Maintaining normal body weight	34.2 (33.8–34.5)	36.5 (35.7–37.2)^¶,^**	35.3 (34.7–36.0)^¶,^**	33.3 (32.7–33.9)^¶,^**	32.9 (32.1–33.7)^¶,^**	30.6 (29.8–1.4)^¶^	28.9 (28.0–9.8)**
Meeting aerobic physical activity recommendations	50.7 (50.4–51.1)	51.4 (50.6–52.2)^¶,^**	51.4 (50.7–52.1)^¶,^**	51.1 (50.5–51.7)^¶,^**	50.7 (49.8–51.5)^¶,^**	49.2 (48.3–50.1)^¶^	46.7 (45.7–7.7)**
Sufficient sleep^††^	62.1 (61.7–62.4)	62.4 (61.6–63.2)	61.7 (61.0–62.4)	62.4 (61.8–63.1)	62.1 (61.2–62.9)	61.1 (60.2–62.0)	61.5 (60.4–62.5)
**No. of health-related behaviors**							
0	1.5 (1.4–1.5)	1.3 (1.1–1.5)	1.5 (1.3–1.7)	1.4 (1.3–1.6)	1.7 (1.5–2.0)	1.7 (1.4–2.0)	1.6 (1.3–1.9)
1	8.6 (8.4–8.8)	7.7 (7.3–8.1)	8.6 (8.2–9.0)	8.7 (8.3–9.0)	9.3 (8.8–9.9)	10.0 (9.4–10.6)	10.3 (9.6–11.0)
2	24.4 (24.1–24.7)	23.8 (23.1–24.5)	24.4 (23.8–24.9)	24.5 (23.9–25.1)	24.6 (23.8–25.4)	24.8 (24.0–25.6)	26.1 (25.2–27.0)
3	35.2 (34.9–35.6)	35.5 (34.7–36.3)	35.4 (34.7–36.0)	35.0 (34.3–35.6)	34.8 (34.0–35.7)	34.8 (33.9–35.6)	35.0 (34.0–36.0)
4	24.1 (23.8–24.4)	25.1 (24.4–25.8)	23.9 (23.4–24.5)	24.3 (23.8–24.8)	23.3 (22.6–24.0)	23.1 (22.4–23.9)	22.0 (21.2–22.9)
5	6.2 (6.1–6.4)	6.7 (6.3–7.0)	6.3 (6.0–6.6)	6.2 (5.9–6.5)	6.3 (5.9–6.7)	5.7 (5.4–6.1)	5.0 (4.6–5.4)

**Abbreviation:** CI = confidence interval.

* Age adjusted to the 2000 U.S. standard population aged ≥18 years with the direct method. The five health-related behaviors are sufficient sleep, current nonsmoking, nondrinking or moderate drinking, maintaining normal body weight, and meeting aerobic leisure time physical activity recommendations.

^†^ As defined in the National Center for Health Statistics 2013 Urban-Rural Classification Scheme for Counties.

^§^ Respondents aged 18–20 years were defined as nondrinking or moderate drinking only if they did not drink alcohol because any alcohol consumption is illegal for those ages.

^¶^ t-test p<0.05 for significant difference between rural respondents and respondents in any other urban-rural classification group.

** t-test p<0.05 for significant difference between micropolitan respondents and respondents in any other urban-rural classification group.

^††^ Sufficient sleep during an average 24-hour period defined as a sleep duration ≥8 hours for persons aged 18–21 years and ≥7 hours for those aged ≥22 years.

In 2013, the overall age-adjusted prevalence of reporting at least four of the five health-related behaviors was 30.4%. The lowest age-adjusted prevalence was observed among adults in noncore counties (27.0%) compared with those living in large metropolitan centers (31.7%), large fringe metropolitan counties (30.2%), medium metropolitan counties (30.5%), small metropolitan counties (29.5%), and micropolitan counties (28.8%) (Table 2). Regardless of urban-rural status, reporting of four or five health-related behaviors occurred more frequently among women than among men; among people aged ≥65 years compared with persons in younger age groups; and among persons with greater educational attainment. Non-Hispanic black adults had lower prevalence of four or five health-related behaviors compared with other race/ethnicity groups for the majority of urban-rural categories. Age-specific prevalence of reporting four or five health-related behaviors was lowest among adults in noncore categories compared with the majority of metropolitan categories, except for adults aged 18–24 years or ≥75 years for whom it did not vary among urban-rural classifications. Typically, age-adjusted prevalence of reporting four or five health-related behaviors was lower in noncore counties compared with other urban-rural classifications among women, men, non-Hispanic whites, Hispanics, and persons with some college education. In contrast, living in metropolitan counties did not provide benefits to non-Hispanic blacks, multiracial non-Hispanics, or American Indian/Alaska Natives; however, Asians living in noncore counties reported four or five health-related behaviors more often than those living in large metropolitan centers. Among adults whose educational attainment was less than high school graduate or high school graduate, those living in either large metropolitan centers or medium metropolitan counties had higher age-adjusted prevalence of reporting four or five health-related behaviors compared with those living in noncore counties.

**TABLE 2:** Prevalence of reporting four or five health-related behaviors* among adults aged ≥18 years, by urban-rural status^†^ and selected demographic characteristics^§^ — Behavioral Risk Factor Surveillance System, United States, 2013

Characteristic	Overall	Large metropolitan center	Large fringe metropolitan	Medium metropolitan	Small metropolitan	Micropolitan	Noncore
	% (95% CI)	% (95% CI)	% (95% CI)	% (95% CI)	% (95% CI)	% (95% CI)	% (95% CI)
**Total**	**30.4 (30.0**–**30.7)**	**31.7 (31.0–32.5)**^¶^^,^**	**30.2 (29.6–30.9)**^¶^^,^**	**30.5 (29.9–31.0)**^¶^^,^**	**29.5 (28.8–30.3)** ^¶^	**28.8 (28.0–29.6)** ^¶^	**27.0 (26.2–27.9)** ^††^
**Sex**							
Men	27.3 (26.9–27.8)	28.8 (27.8–29.8)^¶,^**	26.5 (25.6–27.3)^¶^	27.7 (26.9–28.5)^¶^	26.7 (25.6–27.8)^¶^	26.3 (25.2–27.4)	25.0 (23.8–26.2)
Women	33.5 (33.0–33.9)	34.8 (33.8−35.8)^¶,^**	34.0 (33.1–34.9)^¶,**^	33.3 (32.4–34.1)^¶,^**	32.6 (31.5–33.7)^¶^	31.5 (30.4–32.6)^¶^	29.2 (28.0–30.4)^††^
**Age (yrs)**							
18–24	28.2 (26.9–29.5)	30.9 (28.2–33.8)	27.6 (25.0–30.4)	26.1 (23.7–28.6)	27.0 (23.9–30.4)	27.7 (24.7–31.0)	27.1 (23.3–31.3)
25–34	25.5 (24.7–26.2)	26.9 (25.4–28.6)^¶^	25.7 (24.2–27.3)^¶^	24.7 (23.4–26.1)^¶^	25.1 (23.2–27.1)^¶^	24.1 (22.3–26.1)	20.9 (18.9–23.0)
35–44	27.1 (26.3–27.9)	30.0 (28.2–31.8)^¶,^**	27.1 (25.7–28.6)^¶,^**	26.5 (25.1–27.9)^¶,^**	25.0 (23.2–26.9)^¶^	23.8 (22.1–25.6)	21.8 (20.0–23.8)
45–54	26.4 (25.8–27.1)	28.3 (26.7–29.9)^¶,^**	26.2 (25.1–27.4)^¶,^**	27.4 (26.3–28.6)^¶,^**	24.4 (22.9–26.0)	23.8 (22.3–25.4)	22.5 (20.9–24.2)
55–64	29.9 (29.3–30.5)	30.0 (28.4–31.6)^¶^	30.8 (29.6–32.0)^¶^	30.0 (28.9–31.1)^¶^	29.5 (28.1–31.0)	29.0 (27.6–30.4)	27.6 (26.2–29.1)
65–74	38.0 (37.3–38.8)	37.4 (35.5–39.4)	37.3 (35.9–38.8)	39.5 (38.2–40.8)^¶^	40.0 (38.3–41.7)^¶^	37.5 (35.8–39.1)	36.7 (35.0–38.3)
≥75	47.9 (47.1–48.8)	48.3 (46.0–50.6)	46.7 (44.9–48.4)	49.3 (47.9–50.8)	48.9 (47.0–50.8)	46.7 (44.6–48.8)	47.1 (44.9–49.3)
**Race/Ethnicity**							
White, non-Hispanic	30.9 (30.6–31.3)	33.6 (32.7–34.5)^¶,^**	30.4 (29.7–31.1)^¶,^**	31.3 (30.6–31.9)^¶,^**	30.2 (29.4–31.1)^¶^	29.2 (28.3–30.1)^¶^	27.6 (26.6–28.5)^††^
Black, non-Hispanic	23.4 (22.5–24.3)	23.5 (21.9–25.3)	23.5 (21.7–25.3)	23.7 (21.9–25.6)	21.4 (18.9–24.3)	25.4 (22.2–28.9)	21.1 (18.2–24.4)
Hispanic	28.4 (27.3–29.5)	29.2 (27.4–31.1)^¶^	27.4 (25.2–29.8)^¶^	28.6 (26.8–30.5)^¶^	27.8 (24.7–31.1)^¶^	27.0 (24.0–30.3)	22.0 (18.3–26.2)
American Indian/Alaska Native	26.0 (23.4–28.9)	31.1 (23.3–40.1)	24.1 (18.6–30.5)	24.4 (18.8–31.0)	27.6 (22.4–33.4)	23.3 (19.1–28.1)	25.4 (21.2–30.2)
Asian	42.1 (39.7–44.4)	39.8 (36.3–43.5)^¶^	45.7 (41.5–49.9)	42.1 (37.4–47.0)	45.4 (38.1–52.9)	42.0 (32.6–52.1)	58.2 (43.8–71.3)
Native Hawaiian/Pacific Islander	34.2 (27.4–41.6)	38.2 (28.1–49.4)	40.5 (25.8–57.1)	22.5 (14.2–33.9)	35.5 (22.8–50.5)	21.9 (12.7–35.3)	—^¶^
Multiracial, non-Hispanic	24.5 (22.3–26.8)	26.3 (21.3–32.1)	22.3 (18.4–26.7)	26.3 (22.8–30.2)	21.4 (17.8–25.5)	23.5 (19.5–28.0)	19.4 (15.2–24.4)
Other	30.7 (28.7–32.8)	33.6 (29.2–38.3)	29.5 (25.6–33.7)	32.0 (28.1–36.1)	28.5 (24.3–33.2)	25.6 (21.2–30.5)	27.1 (21.7–33.3)
**Education (yrs)**							
<12	22.5 (21.5–23.7)	24.9 (22.6–27.3)^¶^	21.6 (19.4–24.0)	22.2 (20.2–24.4)^¶^	19.8 (17.4–22.4)	21.1 (18.5–24.0)	18.7 (16.5–21.0)
12	26.3 (25.8–26.9)	27.8 (26.4–29.4)^¶,^**	25.7 (24.4–27.0)	26.6 (25.6–27.7)	25.8 (24.5–27.2)	24.8 (23.5–26.1)	25.0 (23.6–26.5)
>12	33.9 (33.5–34.3)	34.8 (33.9–35.7)^¶^	33.3 (32.6–34.1)^¶^	34.0 (33.2–34.7)^¶^	33.8 (32.8–34.8)^¶^	33.7 (32.7–34.7)^¶^	31.4 (30.3–32.6)^††^

**Abbreviation:** CI= confidence interval.

* The five health-related behaviors are sufficient sleep, current nonsmoking, nondrinking or moderate drinking, maintaining normal body weight, and meeting aerobic leisure time physical activity recommendations.

^†^ As defined in the National Center for Health Statistics 2013 Urban-Rural Classification Scheme for Counties.

^§^ Age adjusted to the 2000 U.S. standard population aged ≥18 years with the direct method, except for age groups.

^¶^ Unreliable estimate if relative standard error ≥ 30% or n < 50.

** t-test p<0.05 for significant difference between rural respondents and respondents in any other urban-rural classification group.

## Discussion

This is the first report to document urban and rural differences in the United States regarding prevalence of selected *Healthy People 2020* health-related behaviors (sufficient sleep, current nonsmoking, nondrinking or moderate drinking, maintaining normal body weight, and meeting aerobic leisure time physical activity recommendations) (https://www.healthypeople.gov/2020/topics-objectives). Because approximately one third of U.S. adults practice at least four of the five behaviors, these findings suggest an ongoing need to increase public awareness and public education, particularly in rural counties where prevalence of these health-related behaviors is lowest. In 2013, although the prevalence of sufficient sleep did not vary by urban-rural classification, none of the six urban-rural categories were close to meeting the *Healthy People 2020* target of 70.8% for sufficient sleep. The tobacco target is to reduce current smoking to 12.0%, which would translate to 88.0% for current nonsmoking; in 2013, the prevalence of current nonsmoking ranged from 74.9% in noncore counties to 83.9% in large metropolitan centers, suggesting that the target is close to being met in more metropolitan populations. In 2013, the more urban categories (large central metropolitan, large fringe metropolitan, and medium metropolitan) met the *Healthy People 2020* target for maintaining normal body weight (33.9%); to achieve the objective, small metropolitan, micropolitan, and noncore counties will require additional obesity prevention efforts. In 2013, the *Healthy People 2020* target for meeting aerobic leisure time physical activity recommendations (47.9%) was attained by populations in metropolitan and micropolitan counties. To achieve the objective, populations in small metropolitan, micropolitan, and noncore counties will require education about the health benefits of aerobic leisure time activity.

Although adults living in noncore counties tended to have lower prevalence of reporting four or five behaviors than those living in metropolitan and micropolitan counties for most of the sociodemographic subgroups, living in metropolitan counties did not provide any benefit to non-Hispanic blacks, American Indian/Alaska Natives, or multiracial non-Hispanics. When compared with other race/ethnicity groups and education subgroups, non-Hispanic blacks, multiracial non-Hispanics, and adults whose educational attainment was less than high school graduate or high school graduate were at a greater disadvantage even in metropolitan counties. For example, lower prevalence of reporting four or five health-related behaviors was observed in the most advantageous category (large central metropolitan) for blacks compared with the most disadvantageous category (noncore) for whites (23.5% versus 27.6%, respectively). Similarly, lower prevalence of reporting four or five health-related behaviors was observed in the most advantageous category for adults whose educational attainment was less than high school graduate or high school graduate compared with the most disadvantageous category for adults with some college education (24.9% and 27.8% versus 31.4%, respectively). Thus, the commonly observed disparities for non-Hispanic blacks and adults with lower educational attainment are even greater in noncore counties than they are in metropolitan counties (*12*). The findings in this report are consistent with studies of the rural penalty defined as the disproportionate rate of mortality in rural areas (*13*).

Regardless of the chosen definition for urban-rural status, health outcomes are consistently poorer in rural areas due in part to a high prevalence of risk factors and to lower socioeconomic status, insurance coverage, and access to quality health care (*2*,*5*–*9*,*12*–*15*). Rural populations are also typically older than the general population. All of these factors lead to longer travel distances to, and higher costs associated with, needed health care services (*2*,*13*,*14*). Major health indicators (e.g., chronic disease, all-cause mortality, dental care, mental health, health insurance coverage, and physician supply) are consistently lower among populations in noncore counties than in micropolitan and metropolitan counties (*2*). These findings are important because 14.8% (46.2 million persons) of the total U.S. population reside in the 63.0% of counties that are classified as either micropolitan or noncore (*10*).

## Limitations

The findings in this report are subject to at least six limitations. First, health-related behaviors in BRFSS are self-reported and subject to reporting and social desirability bias. Second, findings are not generalizable because BRFSS is a household telephone survey; the study population does not include adults who live in long-term care facilities, prisons, and other institutions. Third, state response rates were relatively low and response rates cannot be obtained by urban-rural classifications; therefore, nonresponse might have resulted in either overestimates or underestimates. However, a strength is that BRFSS provides the largest study population with large stable sample sizes for all six urban-rural classifications. Fourth, no data exist about whether social desirability bias differs between rural and nonrural populations. Fifth, moderate drinking was combined with nondrinking to define the alcohol measure in this study, in contrast to the *Healthy People 2020* measure for harmful excessive alcohol use; moderate drinking should not be interpreted as a potential health benefit, which emerging scientific evidence might not support (http://iogt.se/wp-content/uploads/Alkoholrapp-2014_ENG-s%C3%A4rtryck.pdf). The U.S. Dietary Guidelines does not recommend that nondrinkers should start drinking for any reason (https://health.gov/dietaryguidelines/2015/guidelines/appendix-9/). Finally, just as populations in all large central metropolitan counties might not reflect all inner city populations, not all populations in rural counties are the same. Furthermore, large metropolitan counties often include small cities and sparsely populated rural pockets that are located at a considerable distance from the primary urban core (*9*). Variations in influential factors might exist that were not assessed in this study, which might affect health-related behaviors in some but not all rural counties. Such influential factors might include tobacco and alcohol laws and policies, ongoing prevention programs, distance to health care providers, availability of nutritional foods, access to physical activity facilities and opportunities, and commuting times to employment. These factors also might affect the effectiveness and appropriateness of interventions that support adoption of health-related behaviors in rural populations.

## Future Direction

Improving the health of persons living in rural counties might require a more analytically rigorous, multilevel approach that considers the contextual factors that might be mediating the associations between rural status, health-related behaviors, and health and identifies resources and interventions that support the adoption of these behaviors. The Community Preventive Services Task Force has published recommendations for programs and policy interventions that have proven scientific effectiveness for the prevention of excessive alcohol consumption, obesity, physical inactivity, and tobacco use (https://www.thecommunityguide.org/). Additional recommendations and strategies exist for the prevention of obesity (https://www.cdc.gov/obesity/downloads/community_strategies_guide.pdf) and tobacco use (*16*). Some CDC community strategies are being adapted to rural communities for nutrition-related policy and environmental approaches such as food access (*17*).

Further, several similar interventions that are recommended for public health disciplines include those that increase prices (e.g., alcohol, tobacco, and sugar-sweetened beverages) (*8*,*16*,*18*); reduce access (e.g., tobacco and alcohol retail regulations and policies to reduce screen time to increase physical activity) (*19*); and prohibit use in public (e.g., tobacco) (*16*). These interventions should be balanced with general approaches to increasing health-promoting behaviors, such as increasing access to nutritious food or places for physical activity. However, few recommendations focus on strategies to improve rural health (http://www.countyhealthrankings.org/roadmaps/what-works-for-health). Evidence-based policy recommendations do not exist for adult sleep health, which is emerging as an important risk factor for chronic and other health conditions.

Several federal programs address health-related behaviors in rural communities. CDC’s Programs to Reduce Obesity in High Obesity Areas targets counties with obesity rates >40%, many of which include rural communities (https://www.cdc.gov/nccdphp/dnpao/state-local-programs/high-obesity-communities/index.html). Local Foods, Local Places, sponsored by the U.S. Department of Agriculture (USDA), Environmental Protection Agency, CDC, U.S. Department of Transportation, U.S. Department of Housing and Urban Development, White House Rural Council, and regional partners, is an interagency initiative implemented in rural and small communities that aims to improve access to healthy local food (https://www.epa.gov/smartgrowth/local-foods-local-places). USDA’s Community Assessment and Education to Promote Behavioral Health Planning and Evaluation program targets improved community behavioral health in rural counties (https://nifa.usda.gov/program/rural-health-and-safety). Other efforts to support the health of rural communities are funded by CDC’s Division of Nutrition, Physical Activity, and Obesity. These include the Prevention Research Centers (https://www.cdc.gov/PRC/); practitioner program guidance, such as a success stories website that permits searches by filters such as rural communities (https://nccd.cdc.gov/nccdsuccessstories/searchstories.aspx); and the Nutrition and Obesity Research and Evaluation Network, which is an academic research network on rural food access (http://nopren.org/).

Implementation of evidence-based policy interventions and elimination of geographic disparities in policy adoption would improve rural health outcomes. For example, although approximately 50% of the United States population is protected by state and local comprehensive smoke-free laws, no state in the southern United States has a statewide comprehensive smoke-free law (*20*). Furthermore, cigarette excise taxes (https://www.healthypeople.gov/2020/topics-objectives/topic/tobacco-use/objectives) are known to reduce the demand for cigarettes. Southern states, which have many rural counties, have some of the lowest cigarette excise taxes in the country (*21*). Except for large metropolitan counties, information about smoking prevalence and the implementation of evidence-based tobacco control interventions is limited.

## Conclusion

The findings in this report indicate that the prevalence of current nonsmoking, maintaining normal body weight, and meeting aerobic leisure time physical activity recommendations was lowest and the prevalence of nondrinking or moderate drinking was highest in noncore counties. The prevalence of sufficient sleep was low and did not vary by urban-rural classification. The prevalence of having at least four of five health-related behaviors was achieved by less than one third of the U.S. population and was lowest in noncore counties. Chronic disease prevention efforts focus on improving the communities, schools, worksites, and health systems in which persons live, learn, work, and play. Evidence-based strategies to improve the health-related behaviors of persons living in rural areas in the United States should be widely implemented.
