# *Errata*: Vol. 66, No. SS-5

**DOI:** 10.15585/mmwr.mm6607a5

**Published:** 2017-02-24

In the Surveillance Summary “Health-Related Behaviors by Urban-Rural County Classification — United States, 2013,” errors occurred in Table 1 and Table 2. On page 5, in Table 1, under the Micropolitan column, the 95% CI values should have been **(75.7–77.3)** for the current nonsmoking row and **(29.8–31.4)** for the maintaining normal body weight row; under the Noncore column, the 95% CI values should have been **(74.0–75.9)** for the current nonsmoking row, **(28.0–29.8)** for the maintaining body weight row, and **(45.7–47.7)** for the meeting aerobic physical activity recommendations row. On page 6, in Table 2, errors occurred in footnotes and the table is reprinted below.

**TABLE 2:** Prevalence of reporting four or five health-related behaviors* among adults aged ≥18 years, by urban-rural status^†^ and selected demographic characteristics^§^ — Behavioral Risk Factor Surveillance System, United States, 2013

Characteristic	Overall	Large metropolitan center	Large fringe metropolitan	Medium metropolitan	Small metropolitan	Micropolitan	Noncore
	% (95% CI)	% (95% CI)	% (95% CI)	% (95% CI)	% (95% CI)	% (95% CI)	% (95% CI)
**Total**	**30.4 (30.0–30.7)**	**31.7 (31.0–32.5)^¶^**	**30.2 (29.6–30.9)^¶^**	**30.5 (29.9–31.0)^¶^**	**29.5 (28.8–30.3)^¶^**	**28.8 (28.0–29.6)^¶^**	**27.0 (26.2–27.9)^¶^**
**Sex**							
Men	**27.3 (26.9–27.8)**	28.8 (27.8–29.8)**^¶^**	26.5 (25.6–27.3)**^¶^**	27.7 (26.9–28.5)**^¶^**	26.7 (25.6–27.8)**^¶^**	26.3 (25.2–27.4)	25.0 (23.8–26.2)
Women	**33.5 (33.0–33.9)**	34.8 (33.8–35.8)**^¶^**	34.0 (33.1–34.9)**^¶^**	33.3 (32.4–34.1)**^¶^**	32.6 (31.5–33.7)**^¶^**	31.5 (30.4–32.6)**^¶^**	29.2 (28.0–30.4)**^¶^**
**Age (yrs)**							
18–24	**28.2 (26.9–29.5)**	30.9 (28.2–33.8)	27.6 (25.0–30.4)	26.1 (23.7–28.6)	27.0 (23.9–30.4)	27.7 (24.7–31.0)	27.1 (23.3–31.3)
25–34	**25.5 (24.7–26.2)**	26.9 (25.4–28.6)**^¶^**	25.7 (24.2–27.3)**^¶^**	24.7 (23.4–26.1)**^¶^**	25.1 (23.2–27.1)**^¶^**	24.1 (22.3–26.1)	20.9 (18.9–23.0)
35–44	**27.1 (26.3–27.9)**	30.0 (28.2–31.8)**^¶^**	27.1 (25.7–28.6)**^¶^**	26.5 (25.1–27.9)**^¶^**	25.0 (23.2–26.9)**^¶^**	23.8 (22.1–25.6)	21.8 (20.0–23.8)
45–54	**26.4 (25.8–27.1)**	28.3 (26.7–29.9)**^¶^**	26.2 (25.1–27.4)**^¶^**	27.4 (26.3–28.6)**^¶^**	24.4 (22.9–26.0)	23.8 (22.3–25.4)	22.5 (20.9–24.2)
55–64	**29.9 (29.3–30.5)**	30.0 (28.4–31.6)**^¶^**	30.8 (29.6–32.0)**^¶^**	30.0 (28.9–31.1)**^¶^**	29.5 (28.1–31.0)	29.0 (27.6–30.4)	27.6 (26.2–29.1)
65–74	**38.0 (37.3–38.8)**	37.4 (35.5–39.4)	37.3 (35.9–38.8)	39.5 (38.2–40.8)**^¶^**	40.0 (38.3–41.7)**^¶^**	37.5 (35.8–39.1)	36.7 (35.0–38.3)
≥75	**47.9 (47.1–48.8)**	48.3 (46.0–50.6)	46.7 (44.9–48.4)	49.3 (47.9–50.8)	48.9 (47.0–50.8)	46.7 (44.6–48.8)	47.1 (44.9–49.3)
**Race/Ethnicity**							
White, non-Hispanic	**30.9 (30.6–31.3)**	33.6 (32.7–34.5)**^¶^**	30.4 (29.7–31.1)**^¶^**	31.3 (30.6–31.9)**^¶^**	30.2 (29.4–31.1)**^¶^**	29.2 (28.3–30.1)**^¶^**	27.6 (26.6–28.5)**^¶^**
Black, non-Hispanic	**23.4 (22.5–24.3)**	23.5 (21.9–25.3)	23.5 (21.7–25.3)	23.7 (21.9–25.6)	21.4 (18.9–24.3)	25.4 (22.2–28.9)	21.1 (18.2–24.4)
Hispanic	**28.4 (27.3–29.5)**	29.2 (27.4–31.1)**^¶^**	27.4 (25.2–29.8)**^¶^**	28.6 (26.8–30.5)**^¶^**	27.8 (24.7–31.1)**^¶^**	27.0 (24.0–30.3)	22.0 (18.3–26.2)
American Indian/Alaska Native	**26.0 (23.4–28.9)**	31.1 (23.3–40.1)	24.1 (18.6–30.5)	24.4 (18.8–31.0)	27.6 (22.4–33.4)	23.3 (19.1–28.1)	25.4 (21.2–30.2)
Asian	**42.1 (39.7–44.4)**	39.8 (36.3–43.5)**^¶^**	45.7 (41.5–49.9)	42.1 (37.4–47.0)	45.4 (38.1–52.9)	42.0 (32.6–52.1)	58.2 (43.8–71.3)
Native Hawaiian/Pacific Islander	**34.2 (27.4–41.6)**	38.2 (28.1–49.4)	40.5 (25.8–57.1)	22.5 (14.2–33.9)	35.5 (22.8–50.5)	21.9 (12.7–35.3)	—**
Multiracial, non-Hispanic	**24.5 (22.3–26.8)**	26.3 (21.3–32.1)	22.3 (18.4–26.7)	26.3 (22.8–30.2)	21.4 (17.8–25.5)	23.5 (19.5–28.0)	19.4 (15.2–24.4)
Other	**30.7 (28.7–32.8)**	33.6 (29.2–38.3)	29.5 (25.6–33.7)	32.0 (28.1–36.1)	28.5 (24.3–33.2)	25.6 (21.2–30.5)	27.1 (21.7–33.3)
**Education (yrs)**							
<12	**22.5 (21.5–23.7)**	24.9 (22.6–27.3)**^¶^**	21.6 (19.4–24.0)	22.2 (20.2–24.4)**^¶^**	19.8 (17.4–22.4)	21.1 (18.5–24.0)	18.7 (16.5–21.0)
12	**26.3 (25.8–26.9)**	27.8 (26.4–29.4)**^¶^**	25.7 (24.4–27.0)	26.6 (25.6–27.7)	25.8 (24.5–27.2)	24.8 (23.5–26.1)	25.0 (23.6–26.5)
>12	**33.9 (33.5–34.3)**	34.8 (3 3.9–35.7)**^¶^**	33.3 (32.6–34.1)**^¶^**	34.0 (33.2–34.7)**^¶^**	33.8 (32.8–34.8)**^¶^**	33.7 (32.7–34.7)**^¶^**	31.4 (30.3–32.6)**^¶^**

**Abbreviation:** CI = confidence interval.

* The five health-related behaviors are sufficient sleep, current nonsmoking, nondrinking or moderate drinking, maintaining normal body weight, and meeting aerobic leisure time physical activity recommendations.

^†^ As defined in the National Center for Health Statistics 2013 Urban-Rural Classification Scheme for Counties.

^§^ Age adjusted to the 2000 U.S. standard population aged ≥18 years with the direct method, except for age groups.

^¶^ t-test p<0.05 for significant difference between rural respondents and respondents in any other urban-rural classification group.

** Unreliable estimate if relative standard error ≥30% or n <50.

